# Integrative analysis of RNA-Seq data and machine learning approaches to identify Biomarkers for Rhizoctonia solani resistance in sugar beet

**DOI:** 10.1016/j.bbrep.2025.101920

**Published:** 2025-01-19

**Authors:** Bahman Panahi, Mahdi Hassani, Nahid Hosseinzaeh Gharajeh

**Affiliations:** a Department of Genomics, Branch for Northwest & West Region, Agricultural Biotechnology Research Institute of Iran (ABRII), Agricultural Research, Education and Extension Organization (AREEO), Tabriz, 5156915-598, Iran; b Sugar Beet Seed Institute (SBSI), Agricultural Research, Education and Extension Organization (AREEO), Karaj, Iran

**Keywords:** Crown and root rot, Sugar beet, Biomarker, Machine-learning

## Abstract

Rhizoctonia solani is a significant pathogen that causes crown and root rot in sugar beet (Beta vulgaris), leading to considerable yield losses. To develop resilient cultivars, it is crucial to understand the molecular mechanisms underlying both resistance and susceptibility. In this study, we employed RNA-Seq analysis alongside machine learning techniques to identify key biomarkers associated with resistance to R. solani. We ranked differentially expressed genes (DEGs) using feature-weighting algorithms, such as Relief and kernel-based methods, to model expression patterns between sensitive and tolerant cultivars. Our integrative approach identified several candidate genes, including Bv5g001004 (encoding Ethylene-responsive transcription factor 1A), Bv8g000842 (encoding 5′-adenylylsulfate reductase 1), and Bv7g000949 (encoding Heavy metal-associated isoprenylated plant protein 5). These genes are involved in stress signal transduction, sulfur metabolism, and disease resistance pathways. Graphical visualizations of the Random Forest and Decision Tree models illustrated the decision-making processes and gene interactions, enhancing our understanding of the complex relationships between sensitive and tolerant genotypes. This study demonstrates the effectiveness of integrating RNA-Seq and machine learning techniques for biomarker discovery and highlights potential targets for developing R. solani-resistant sugar beet cultivars. The findings provide a robust framework for improving crop enhancement strategies and contribute to sustainable agricultural practices by increasing stress resilience in economically important crops.

## Highlights

• Bv5g001004, Bv8g000842, and Bv7g000949 were proposed as potential Biomarkers for Rhizoctonia resistance sugar beet.
• The efficiency of integrative approach for biomarker prediction in sugar beet was highlighted.
• The current study enhanced our understanding of the complex relationships between sensitive and tolerant genotypes.

## 1 Introduction

Sugar beet (*Beta vulgaris*) is a crucial crop for global sugar production, significantly contributing to the food, biofuel, and livestock feed industries. However, its cultivation faces challenges from *Rhizoctonia solani*, a soil-borne fungal pathogen responsible for Rhizoctonia root rot, which leads to considerable yield losses and decreased crop quality [1]. To mitigate the impact of this pathogen and ensure sustainable production, it is essential to develop sugar beet varieties resistant to *R. solani* [2].

Generally, a comprehensive understanding of the molecular mechanisms is associated with search of candidate biomarkers. The omics methods help with this important task [3,4]. Among different omics methods, RNA sequencing (RNA-Seq), have proven invaluable for studying plant-pathogen interactions [5]. These techniques reveal gene expression changes during pathogen infection and help identify potential resistance genes and the complex regulatory networks involved in disease defense [6]. Despite significant advances in transcriptomic research, challenges remain in fully understanding the genetic basis of *R. solani* resistance in sugar beet [7].

Previous studies have often been limited by small sample sizes, insufficient data integration, and traditional analysis methods that struggle to capture the intricate, non-linear relationships between genes and resistance pathways [8]. Consequently, identifying reliable biomarkers for *R. solani* resistance remains a significant hurdle. Moreover, most research has focused on individual genes or specific pathways, rather than adopting a systems-level approach that encompasses the entire genetic network influencing resistance [9]. There is an urgent need for more advanced techniques, such as machine learning, to enhance data analysis and improve the identification of meaningful biomarkers associated with disease resistance.

In response to these challenges, this study aims to identify biomarkers linked to *R. solani* resistance in sugar beet by integrating RNA-Seq data with machine learning approaches. By leveraging the capabilities of high-throughput RNA sequencing and advanced machine learning techniques, this research seeks to uncover key genes, and biomarkers involved in resistance mechanisms. Machine learning facilitate the identification of complex, non-linear interactions within the data, resulting in a more accurate and comprehensive model of *R. solani* resistance. This study aimed to enhance the understanding of resistance in sugar beet and establish a foundation for developing resistant cultivars through improved biomarker identification.

## 2 Methods and material

### 2.1 Eligible data selection

RNA-sequencing dataset related to Rhizoctonia root-rot diseases in sugar beet (*Beta vulgaris* ssp. *vulgaris)* was obtained from the Sequence Read Archive (SRA) databases. Specifically, we chose dataset PRJEB40905, which includes two partially resistant sugar beet breeding lines (G1: line no. 11014044 09; G2: line no. 06012609 70) and two susceptible lines (G3: line no. 11014038 09; G4: line no. 11014072 09). In this data set, briefly, after a 13-week growth period, the plants were inoculated with the *R. solani* AG2-2IIIB BBA 69670 isolate. The inoculated plants were then transferred from an 18/12 °C (day/night) temperature regime to a 24/18 °C environment to promote the infection phase. Roots from at least three plants per genotype were collected at three time points: prior to infection (day 0), and at 2 and 5 day's post-infection (dpi) as demonstrated. This experimental design was informed by a pilot study, which showed that the fungus reaches the root by 2 dpi and is in its initial phase of infection by 5 dpi. This setup was selected to enhance the data on fungal-induced gene expression while minimizing the impact of genes associated with plant development. The roots were washed, and four samples were collected from each root using a core drill. Additionally, control samples were collected by harvesting four roots from each line before inoculation [8].

### 2.2 RNA seq data preprocessing

The raw FASTQ files for the datasets mentioned were retrieved, and the quality of the short reads was assessed using FastQC software (version 0.11.5) [10]. Reads with quality scores below 30 were removed using Trimmomatic software (version 0.32) with the following parameters: LEADING: 30, TRAILING: 3, SLIDINGWINDOW: 4:20, and MINLEN: 45 [11]. The resulting high-quality paired-end reads, which met the quality criteria, were aligned to the sugar beet genome Ref-Beet-1.0/Dec 2011 scaffold assembly of KWS232 using TopHat (version 2.0.12) [12]. Finally, HTSeq software was employed to quantify the number of mapped reads for each gene.

### 2.3 Differentially expressed genes (DEG)

The purpose of normalization is to scale and standardize data from different samples, allowing for consistent comparison without the influence of variations in the original measurement units or scales. To accomplish this, the count data was normalized using the variance-stabilizing transformation method implemented in DESeq2. Differential expression analysis was conducted using the Bioconductor DESeq2 package (v1.10.1) [13], employing Wald's test to evaluate log2-fold changes. Genes with a coefficient of variation (CV) higher than 10 % were included in study. Ultimately, differentially expressed genes (DEGs) were selected based on the log2-fold change |≥ 1.0| and an adjusted p-value <0.05 criteria.

### 2.4 Feature selection

Feature selection involves identifying a key subset of features—specifically, genes in this context—to enhance predictive model performance. By reducing the number of input variables and eliminating irrelevant or redundant features, we aimed to improve model efficiency. In this study, we applied feature selection to the common differentially expressed gene set using Relief Statistic, as detailed in Ref. [14,15]. The Relief algorithm is a widely used feature selection method in machine learning, designed to identify and rank features based on their relevance to predicting target variables. The Relief algorithm's core principle is to evaluate the relevance of features by examining the differences in feature values between a selected instance and its nearest neighbors. It specifically analyzes the nearest neighbors from the same class (near-hits) and those from different classes (near-misses). The weight for each feature is updated using the following formula:

$$W_{i}=W_{i}-\frac{diff\left(x_{i},x_{near-hit}\right)}{k}+\frac{diff\left(x_{i},x_{near-miss}\right)}{k}$$

Where, $W_{i}$ is the weight assigned to feature i., $diff\left(x_{i},x_{near-hit}\right)$ is the absolute difference between the values of feature i for the current instance and its nearest neighbor from the same class (near-hit). $diff\left(x_{i},x_{near-miss}\right)$ is the absolute difference between the values of feature i for the current instance and its nearest neighbor from a different class (near-miss). K is a user-defined parameter representing the number of nearest neighbors considered.

### 2.5 Model optimization

In this study, we applied three widely used machine learning algorithms—Random Forest, Support Vector Machine (SVM), and Naive Bayes—to develop predictive models that accurately identify key features and patterns within the dataset.

Random Forest is a robust ensemble learning algorithm commonly utilized for classification and regression tasks. It functions by constructing multiple decision trees during the training process and aggregating their predictions to enhance accuracy and reduce the risk of over fitting [16]. The algorithm employs bagging (Bootstrap Aggregating), wherein each tree is trained on a random subset of the data sampled with replacement, ensuring diversity among the trees. Furthermore, a random subset of features is considered at each node split, which further strengthens the model's robustness and mitigates excessive dependence on specific features. For classification tasks, the final prediction of the Random Forest is determined through majority voting among the predictions of n decision trees, as expressed by:

$$y=mode\left(T_{1}\left(x\right),T_{2\;}\left(x\right),\ldots ,T_{n\;}\left(x\right)\right)$$

Here, y is the predicted class, $T_{i\;}\left(x\right)$ represents the prediction of the $i^{th}$ tree, and n donates the total number of trees in the forest. The models were run with a minimal size of two for all leaves, a minimal gain of 0.1 to produce a split, and a maximal tree depth of 20. A confidence level of 0.25 was selected for the pessimistic error calculation for pruning.

The Support Vector Machine (SVM) is a supervised machine learning algorithm widely utilized for both classification and regression tasks. It demonstrates particular effectiveness in high-dimensional spaces and is adept at handling data that is not linearly separable, employing kernel methods to transform the feature space [17]. The core principle of SVM involves identifying the optimal hyperplane that maximally separates data points belonging to distinct classes within the feature space.

Naive Bayes is a straightforward yet effective probabilistic machine learning algorithm commonly employed for classification tasks. It is based on Bayes' Theorem, which facilitates the calculation of the posterior probability of a class based on observed features. The algorithm operates under the assumption that features are conditionally independent given the class label—an assumption considered "naive" that simplifies calculations but frequently produces satisfactory results in practice. The formula for computing the probability of a class label given the features can be derived from Bayes' theorem as follow:

$$p\left(C_{k}\;|\;x_{1\;},x_{2},\ldots ..,x_{n\;}\right)=\frac{P\;\left(x_{1\;},x_{2},\ldots ..,x_{n}|C_{k}\right).P\left(C_{k}\right)}{P\left(x_{1},x_{2},\ldots .,x_{n}\right)}$$

where, $p\left(C_{k}\;|\;x_{1\;},x_{2},\ldots ..,x_{n\;}\right)$ is the probability of class $C_{k}$ given the features $x_{1\;},x_{2},\ldots ..,x_{n}$. $P\;\left(x_{1\;},x_{2},\ldots ..,x_{n}|C_{k}\right)$ is the likelihood of observing the features given class $C_{k}$. $P\left(C_{k}\right)$ is the prior probability of class $C_{k}$, and $P\;\left(x_{1\;},x_{2},\ldots ..,x_{n}|C_{k}\right)$ is the probability of observing the features

### 2.6 Performance evaluation

To assess the performance of the models, we utilized 10-fold cross-validation. This widely used validation technique offers a reliable estimate of model performance by dividing the dataset into ten equal subsets, or folds. In each iteration, one fold is used as the validation set while the remaining nine folds are combined to create the training set. This process is repeated ten times, ensuring that each fold serves as the validation set once, allowing every data point to be used for both training and validation. Mathematically, the overall performance of the model is calculated as the average of the performance metrics (e.g., accuracy, precision, recall, or mean squared error) obtained from each fold. If $M_{i}$ denotes the performance metric for the $i^{th}$ fold, the final performance metric is given by:

$$\bar{M}=\frac{1}{k}\;\sum_{i=1}^{k}M_{i}$$

Where, k=10(numberoffolds), and $M_{i}$ is the metric computed for the $i^{\text{th}}$ fold.

### 2.7 Functional impact analysis

To unveil the functional impact of the identified biomarkers and core DEGs, systems level analysis of protein-protein interactions was performed using network analysis. To carried out such analysis, PPI network were constructed string STRING database using the knowledge extracted from the text mining, experimentally validated data, and co-expression network, with the default median confidence level [18]. Moreover, functional enrichment in systems level analysis was performed in biological process category with a False Discovery Rate (FDR) correction higher than 0.05 as described by Ref. [19].

## 3 Results

### 3.1 Identification of DEGs

**Fig. 1:**
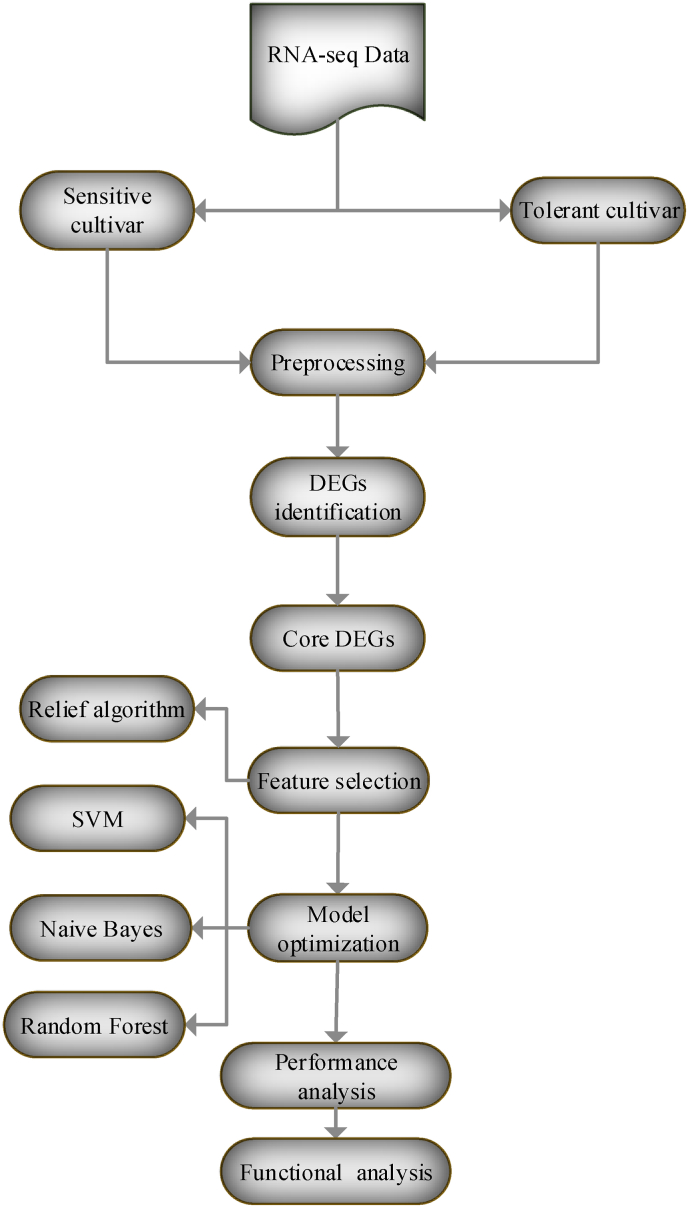
A schematic representation of the comprehensive workflow implemented in this study.

**Fig. 2:**
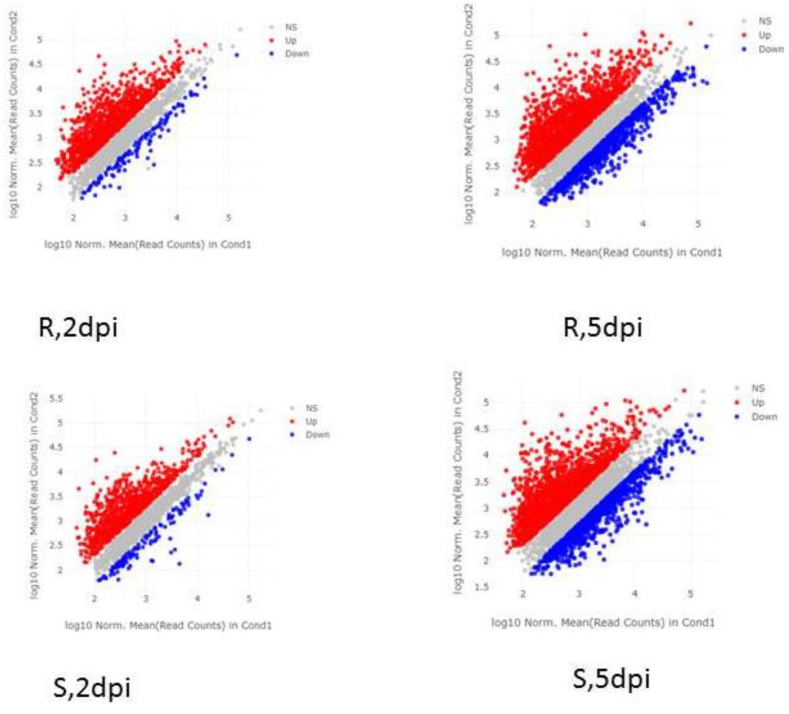
Differentially expressed genes (DEGs) identified in various sugar beet cultivars under different levels of *Rhizoctonia solani* infection. The red spots represent up-regulated genes, while the blue spots indicate down-regulated genes in response to the infection. This visualization illustrates the unique transcriptional responses among cultivars and highlights genes that may be linked to resistance or susceptibility to *R. solani*.

**Fig. 3:**
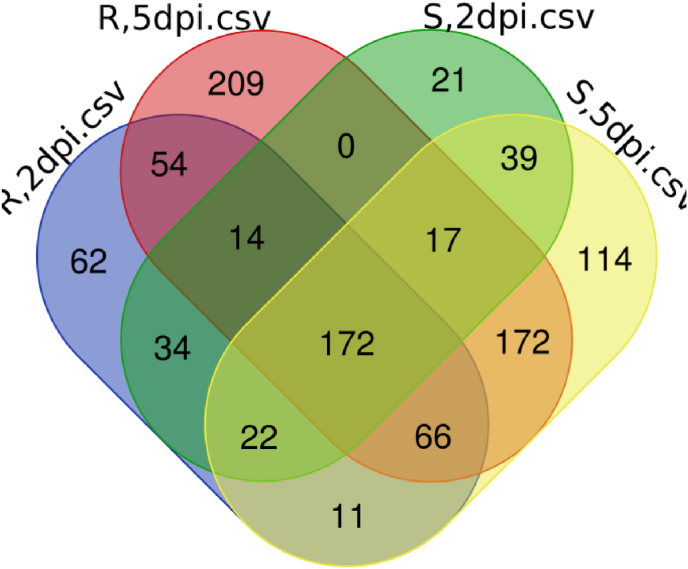
The Venn diagram illustrates the overlap of differentially expressed genes (DEGs) across four treatments. This analysis highlights both shared and unique DEGs among the datasets, offering insights into conserved and condition-specific gene expression patterns. The numbers in the overlapping regions indicate DEGs that are common to multiple datasets, while the numbers in the non-overlapping regions represent DEGs that are unique to a specific dataset.

The outline of the study was presented in Fig. 1. As demonstrated in the methods and material section, differential gene expression (DGE) analysis conducted on sensitive and tolerant cultivars subjected to two distinct concentrations of fungal infection revealed varying numbers of differentially expressed genes (DEGs). As shown in Fig. 2, the analysis identified 319 DEGs in the sensitive cultivar when infected at 2 days post-inoculation (dpi) with the fungal pathogen, this increased to 613 DEGs when the infection was sustained at 5 dpi. In contrast, the tolerant cultivar exhibited 432 DEGs at 2 dpi and 704 DEGs at 5 dpi under the same fungal treatment conditions. A comparative analysis of these DEGs highlighted 172 genes, which was designed as a core DEGs that were consistently expressed across both cultivars and both infection levels (Fig. 3). We created an expression matrix with these core DEGs. This refined matrix was used for feature selection and model optimization, as outlined in the Methods and Materials section. This refined matrix served as the basis for feature selection and model optimization, as detailed in the Methods and Materials section. By utilizing this enhanced dataset, we identified key features that influence the differing responses of the cultivars. This approach not only improved the predictive models but also increased the accuracy and robustness of the analysis, leading to a more reliable identification of gene expression patterns related to fungal resistance and tolerance.

### 3.2 Functional impact of core DEGs

**Fig. 4:**
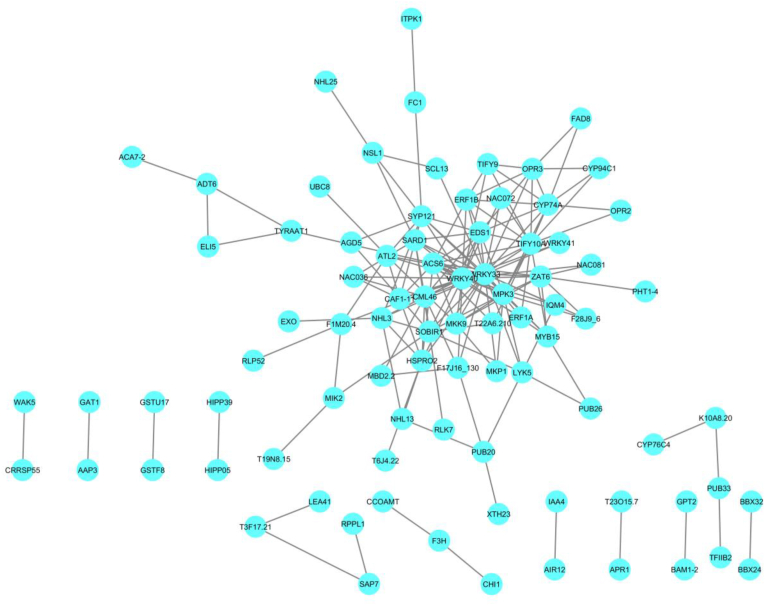
Protein-protein interaction (PPI) network constructed for the core differentially expressed genes (DEGs) identified in sugar beet infected by *Rhizoctonia solani*. This network illustrates the interactions among key proteins encoded by the core DEGs, offering insights into the functional relationships and biological processes related to resistance mechanisms. In this diagram, nodes represent proteins, while edges indicate the interactions between them.

The enriched biological processes among identified core differentially expressed genes (DEGs) in sugar beet in response to *Rhizoctonia solani* infection at systems level highlight critical pathways related to stress adaptation, defense mechanisms, and metabolic reprogramming (Fig. 4). Key processes such as "Response to jasmonic acid" (FDR = 7.95E-7), "Response to oxidative stress" (FDR = 3.98E-5), and "Response to salicylic acid" (FDR = 7.66E-5) demonstrate the activation of essential stress signaling pathways that facilitate defense and mitigate oxidative damage caused by the infection. Additionally, hormonal signaling pathways, including "Response to ethylene" (FDR = 4.1E-4) and "Regulation of jasmonic acid-mediated signaling pathway" (FDR = 7.0E-3), indicate the role of hormonal crosstalk in regulating plant responses to pathogen attack. The significant enrichment in "Regulation of defense response" (FDR = 7.6E-5) and "Positive regulation of defense response" (FDR = 1.07E-2) underscores the involvement of core DEGs in coordinating immune responses. The metabolic reprogramming of sugar beet in response to infection is further reflected in enriched processes such as "Indole-containing compound metabolic process" (FDR = 5.57E-6), "Secondary metabolite biosynthetic process" (FDR = 7.6E-5), and "Aromatic amino acid family metabolic process" (FDR = 9.95E-5), highlighting the significance of secondary metabolites and aromatic compounds in pathogen defense (Table 1). Developmental reprogramming in response to pathogen attack is indicated by processes such as "Anatomical structure development" (FDR = 1.2E-3) and "System development" (FDR = 2.29E-2). Additionally, processes such as "Cellular response to stress" (FDR = 1.7E-3) and "Positive regulation of cell death" (FDR = 4.62E-2) suggest a delicate balance between survival mechanisms and programmed cell death. Broader cellular responses, including "Signal transduction" (FDR = 2.98E-2), "Regulation of cellular process" (FDR = 4.84E-2), and "Cellular response to endogenous stimulus" (FDR = 8.8E-3), emphasize the complex regulatory networks activated in response to the infection. Systemic responses, such as "Induced systemic resistance" (FDR = 3.28E-2), and adaptive processes like "Response to osmotic stress" (FDR = 3.28E-2) and "Response to water deprivation" (FDR = 4.64E-2), suggest broader physiological adjustments beyond the infection site (Table 1). Collectively, these findings illustrate the multifaceted roles of core DEGs in sugar beet's response to *R. solani* infection, encompassing hormonal regulation, defense activation, metabolic shifts, and systemic resistance mechanisms. These insights provide a foundation for the development of targeted strategies to enhance sugar beet's resilience against this pathogen.

**Table 1:** Significantly enriched Gene Ontology (GO) terms associated with DEGs in sugar beet under *Rhizoctonia solani* infection. The table presents the enriched biological processes along with their corresponding false discovery rate (FDR).

GO ID	GO description	False discovery rate
GO:0009753	Response to jasmonic acid	0.000000795
GO:0009719	Response to endogenous stimulus	0.00000282
GO:0042430	Indole-containing compound metabolic process	0.00000557
GO:0006979	Response to oxidative stress	0.0000398
GO:0031347	Regulation of defense response	0.000076
GO:0044550	Secondary metabolite biosynthetic process	0.000076
GO:0009751	Response to salicylic acid	0.0000766
GO:0009072	Aromatic amino acid family metabolic process	0.0000995
GO:0042435	Indole-containing compound biosynthetic process	0.0001
GO:0071456	Cellular response to hypoxia	0.00013
GO:0010120	Camalexin biosynthetic process	0.00015
GO:0009404	Toxin metabolic process	0.00018
GO:0048583	Regulation of response to stimulus	0.0002
GO:0009723	Response to ethylene	0.00041
GO:0042343	Indole glucosinolate metabolic process	0.00063
GO:0051716	Cellular response to stimulus	0.00093
GO:0008219	Cell death	0.0011
GO:0048856	Anatomical structure development	0.0012
GO:0006790	Sulfur compound metabolic process	0.0015
GO:0009873	Ethylene-activated signaling pathway	0.0016
GO:0033554	Cellular response to stress	0.0017
GO:0009759	Indole glucosinolate biosynthetic process	0.0024
GO:0044272	Sulfur compound biosynthetic process	0.0024
GO:0009620	Response to fungus	0.005
GO:0071310	Cellular response to organic substance	0.005
GO:1901605	Alpha-amino acid metabolic process	0.005
GO:0009791	Post-embryonic development	0.0057
GO:0009626	Plant-type hypersensitive response	0.006
GO:0046394	Carboxylic acid biosynthetic process	0.0067
GO:0044283	Small molecule biosynthetic process	0.007
GO:2000022	Regulation of jasmonic acid mediated signaling pathway	0.007
GO:0002831	Regulation of response to biotic stimulus	0.0073
GO:0001101	Response to acid chemical	0.0074
GO:0009416	Response to light stimulus	0.0076
GO:0043436	Oxoacid metabolic process	0.0076
GO:0032101	Regulation of response to external stimulus	0.0081
GO:0071495	Cellular response to endogenous stimulus	0.0088
GO:0009755	Hormone-mediated signaling pathway	0.0097
GO:0006520	Cellular amino acid metabolic process	0.0098
GO:0032501	Multicellular organismal process	0.0104
GO:0010618	Aerenchyma formation	0.0107
GO:0031349	Positive regulation of defense response	0.0107
GO:0009863	Salicylic acid mediated signaling pathway	0.0121
GO:0009695	Jasmonic acid biosynthetic process	0.0135
GO:0019438	Aromatic compound biosynthetic process	0.0135
GO:1901698	Response to nitrogen compound	0.0135
GO:0071407	Cellular response to organic cyclic compound	0.0139
GO:0010243	Response to organonitrogen compound	0.0143
GO:0009862	salicylic acid mediated signaling pathway	0.0147
GO:0010200	Response to chitin	0.0147
GO:0009073	Aromatic amino acid family biosynthetic process	0.0158
GO:0010337	Regulation of salicylic acid metabolic process	0.0162
GO:1901362	Organic cyclic compound biosynthetic process	0.0162
GO:0019752	Carboxylic acid metabolic process	0.0164
GO:0050829	Defense response to Gram-negative bacterium	0.0175
GO:0120254	Olefinic compound metabolic process	0.0191
GO:0009987	Cellular process	0.0207
GO:0048731	System development	0.0229
GO:1901701	Cellular response to oxygen-containing compound	0.0261
GO:1901564	Organonitrogen compound metabolic process	0.0271
GO:0007165	Signal transduction	0.0298
GO:0009640	Photomorphogenesis	0.0298
GO:1900378	Positive regulation of secondary metabolite biosynthetic process	0.0298
GO:1902065	Response to l-glutamate	0.0298
GO:0009627	Systemic acquired resistance	0.0308
GO:0006970	Response to osmotic stress	0.0328
GO:0009682	Induced systemic resistance	0.0328
GO:0031408	Oxylipin biosynthetic process	0.0328
GO:0010035	Response to inorganic substance	0.034
GO:2000031	Regulation of salicylic acid mediated signaling pathway	0.0348
GO:0080151	Positive regulation of salicylic acid mediated signaling pathway	0.0364
GO:0010193	Response to ozone	0.0371
GO:0006568	Tryptophan metabolic process	0.0433
GO:0010942	Positive regulation of cell death	0.0462
GO:0009414	Response to water deprivation	0.0464
GO:0044281	Small molecule metabolic process	0.0466
GO:0050794	Regulation of cellular process	0.0484

### 3.3 Feature selection

Core DEGs were processed through data cleaning to remove useless and highly correlated attributes based on following criteria: variation less than 0.1 and correlation exceeding 95 %. After cleaning, the data were normalized and analyzed using relief algorithm for feature selection. Core DEGs with weighting values above 0.5 were included in further predictive model optimization. This analysis prioritized several key genes including BVRB_7g161540, BVRB_3g056660, BVRB_5g106750, BVRB_6g148820, BVRB_1g010560, encoding Heavy metal-associated isoprenylated plant protein 5 (HIPP05), Mitogen-activated protein kinase kinase 9 (MMK9), N-hydroxycinnamoyl/benzoyltransferase-like protein (MBD2.2), DNA ligase-like protein (DUF1645), Ethylene-responsive transcription factor 1A (ERF1A), and Magnesium dechelatase SGRL (SGRL), respectively.

### 3.4 Model optimization

**Fig. 5:**
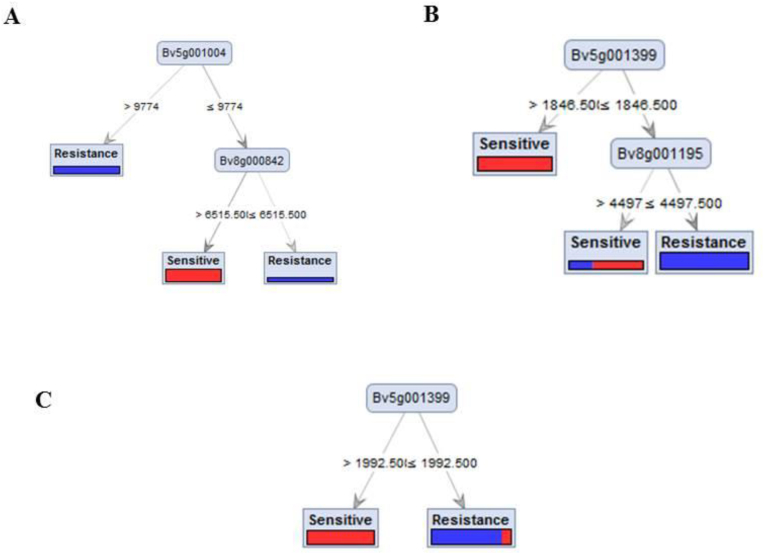
The graphical representation displays core differentially expressed genes (DEGs) based on optimized models. Panel (A) ranks the DEGs using Random Forest with Gain Ratio criteria, panel (B) ranks them using Random Forest with Accuracy criteria, and panel (C) illustrates the DEGs ranked by Decision Tree based on Gini Index criteria. Each panel emphasizes the most important genes identified by the respective algorithms, offering insights into the gene contributions that distinguish resistant and sensitive sugar beet cultivars under *Rhizoctonia solani* infection.

The key genes chosen with feature selection were used for model optimization. Twelve predictive models were constructed using three modeling algorithms with four criteria including information gain ratio, information gain, Gini index and accuracy criteria. Table 2 compares the performance of various algorithms in predicting the sensitivity and tolerance of sugar beet genotypes to *Rhizoctonia* using class recall, class precision, and overall performance. Class recall indicates the percentage of cases correctly identified as either resistant or sensitive. All models achieved a recall of 75 % for resistant genotypes, while Random Forest, Naïve Bayes, and SVM achieved perfect recall of 100 % for sensitive genotypes. In contrast, the Decision Tree recorded a lower recall of 66.67 % for sensitive genotypes. Class precision evaluates the accuracy of predictions for resistant and sensitive genotypes. Random Forest, Naïve Bayes, and SVM attained 100 % precision for resistant genotypes and 75 % for sensitive genotypes. The Decision Tree performed slightly lower, with 75 % precision for resistant genotypes and 66.67 % precision for sensitive genotypes. Overall performance reflects the combined effectiveness of the models. Random Forest, Naïve Bayes, and SVM achieved the highest overall performance at 85.71 %, while the Decision Tree scored lower at 71.43 %. Graphical visualization of optimized models based on Random forest and Decision tree algorithms are shown in Fig. 5.

**Table 2:** Prediction performance of the tolerance and sensitive cultivars based on different algorithms. Precision refers to the number of true positives divided by the total number of positive predictions. Class recall was calculated dividing the number of true positives by the number of positive instances.

Algorithms	Criteria	Class recall (%) (Resistance)	Class recall (%) (Sensitive)	Class precision (%) (Resistance)	Class precision (%) (Sensitive)	Performance (%)
Random Forest	Gain Ratio	75.00	100.00	100.00	75.00	85.71
Decision tree	Accuracy	75.00	66.67	75.00	66.67	71.43
Naïve Bayes	–	75.00	100.00	100.00	75.00	85.71
SVM (Kernel)	–	75.00	100.00	100.00	75.00	85.71

## 4 Discussion

In this study, machine learning (ML) techniques were effectively employed to identify and prioritize biomarker genes in sugar beet (*Beta vulgaris* L.) that differentiate between genotypes sensitive and tolerant to *Rhizoctonia solani*. ML has gained recognition as a powerful tool for biomarker discovery, enabling the analysis of complex datasets and the identification of patterns that traditional statistical methods may overlook [20]. The analysis utilized weighting algorithms, including Relief and kernel-based methods, to evaluate the significance of gene expression features and model their differential patterns across two contrasting sugar beet (*Beta vulgaris*) cultivars. By assigning weights to differentially expressed genes (DEGs) based on their impact on model performance, this approach identified a crucial set of genes that distinguish sensitive and tolerant genotypes under *R. solani* infection.

Among the prioritized genes, Bv5g001004, which encodes Ethylene-responsive transcription factor 1A (ERF1A), acts as a transcriptional activator by binding to the GCC-box in pathogenesis-related promoter elements. ERF1A is vital for regulating gene expression during stress and modulating components of stress signaling pathways, establishing it as a key regulator in the defense response [21]. Its function underscores the importance of ethylene-mediated signaling in activating downstream defense mechanisms against pathogens. Bv7g000949, which encodes Heavy metal-associated isoprenylated plant protein 5 (HIPP05), is a heavy-metal-binding protein linked to disease resistance. As a member of the HIPP family, it likely contributes to managing oxidative stress and maintaining cellular homeostasis during pathogen attacks, reinforcing its role in resilience mechanisms [22]. Bv8g000842, which encodes 5′-adenylylsulfate reductase 1 (APR1), catalyzes the reduction of sulfate to cysteine in the chloroplast, favoring adenosine-5′-phosphosulfate (APS) over 3′-phosphoadenosine-5′-phosphosulfate (PAPS). This enzyme is essential for sulfur assimilation, a critical process in synthesizing sulfur-containing defense compounds. APR1's reliance on glutathione or DTT as proton donors links it directly to the cellular redox state, a key element in stress responses [23]. These genes, along with others like Bv6g004835 (encoding a DNA ligase-like protein), Bv5g001399 (encoding an N-hydroxycinnamoyl/benzoyltransferase-like protein), and Bv3g001159 (encoding Mitogen-activated protein kinase kinase 9, MKK9), form a complex network of signaling and metabolic pathways that collectively mediate the sugar beet's defense against *R. solani*. The inclusion of genes such as Bv7ug004845 (encoding B-box zinc finger protein 32, BBX32) and Bv1g001598 (encoding Magnesium dechelatase SGRL, involved in chlorophyll degradation) further highlights the diverse biochemical and physiological processes that underpin stress adaptation.

Graphical visualizations of optimized machine learning models, such as Random Forest and Decision Tree, have proven invaluable in clarifying the gene interactions and decision-making processes related to Rhizoctonia crown and root rot resistance in sugar beet. These visual tools enhance our ability to interpret high-dimensional gene expression data, simplifying the complex relationships between sensitive and tolerant genotypes. Combining these visualizations with machine learning-based feature ranking not only helps identify critical biomarkers but also provides actionable insights for breeding programs. The Random Forest model, in particular, has emerged as a powerful tool for prioritizing genes based on their contributions to stress tolerance. Key biomarkers identified include Bv5g001004, which encodes Ethylene-responsive transcription factor 1A, and Bv8g000842, which encodes 5′-adenylylsulfate reductase 1. These genes play crucial roles in stress signal transduction and sulfur assimilation, both of which are central to stress adaptation [24]. These findings align with and build upon previous research that emphasizes the significance of ethylene signaling and sulfur metabolism in plant defense against biotic stresses.

## 5 Conclusion

In conclusion, this study highlights the effectiveness of machine learning (ML) techniques in identifying and prioritizing biomarker genes that distinguish between *Rhizoctonia solani*-tolerant and sensitive sugar beet genotypes. By utilizing advanced algorithms, the analysis identified key genes like Bv5g001004 (ERF1A) and Bv8g000842 (APR1), which play crucial roles in ethylene-mediated signaling and sulfur assimilation—both vital for stress adaptation. These findings create a solid foundation for understanding complex gene interactions and developing targeted breeding strategies to improve sugar beet resilience.

The broader implications of this study emphasize the transformative potential of ML in agricultural genomics, allowing for the analysis of high-dimensional datasets and the prioritization of actionable biomarkers. The methodology employed, which integrates feature-ranking algorithms with visual model interpretations, presents a scalable approach that can be applied to other crops and stress conditions. Future research should focus on the functional validation of the identified biomarkers and the incorporation of these findings into genomic selection pipelines, thereby accelerating the development of stress-tolerant cultivars. This work not only contributes to sustainable agriculture but also underscores the importance of computational approaches in addressing global food security challenges.

## Author contributions

BP conceived, designed, and conducted the bioinformatics analyses and wrote the article. MH contribute in bioinformatics analyses and written of the article.

## Data availability

The datasets analyzed during the current study are available in the European Nucleotide Archive (ENA) section of NCBI (https://www.ebi.ac.uk/ena/browser/) repository, with accession number PRJEB40905.

## Declaration of competing interest

The authors declare that there is not any conflict of interest.
